# Predictors of Lymphatic Complications Following Renal Transplant: A Prospective Study Involving Predominantly Living Donor Transplants From India

**DOI:** 10.7759/cureus.17133

**Published:** 2021-08-12

**Authors:** Nitin Agarwal, Gyan R Kumar, Anil K Singh Rana, Abdul Mubeen, Manoj K Dokania

**Affiliations:** 1 Transplant Unit, Department of Surgery, Atal Bihari Vajpayee Institute of Medical Sciences and Ram Manohar Lohia Hospital, Delhi, IND

**Keywords:** lymphocele, lymphatic complications, prolonged drainage, risk factors, living donor renal transplant, prospective

## Abstract

Introduction

Lymphatic complications (LC) are common (up to 33%) and troublesome after renal transplantation. Different studies have established varying medical and surgical risk factors, mostly by retrospective analysis on deceased donor renal transplants (DDRTs). The end-point is mostly lymphocele, with few reports documenting the equally important lymphorrhea.

Methods

In our prospective analytical study done over three years, most were living donor renal transplant (LDRT) pairs by a single team. The primary outcome measure was lymphocele and/or prolonged drainage for more than 15 days, with a six-month follow-up. The variables recorded were age, gender, hemodialysis duration, etiology, relationship, human leucocyte antigen (HLA) mismatch, induction regimen, acute rejection, warm ischemia time (WIT), and delayed graft function (DGF). Univariate analysis was by chi-square and t-tests as applicable, while logistic regression (both simultaneous and forward stepwise) was used for risk factor prediction.

Results

Eligible cases were 150, with 145 (97%) LDRT pairs. Donors were mostly female (122/150; 81%) with mean age (~43 years) higher than recipient age (~33 years). The common etiologies were diabetes (31%), hypertension (23%), and IgA nephropathy (11%). Most donors were mothers (37%) and wives (31%), and 28% of LDRT pairs had HLA mismatch >3. Mean duration of hemodialysis was about 18 months, and mean WIT was 52 minutes. Both DGF (B coefficient= -1.69, p<0.000) and WIT (B=-0.038, p=0.024) were significant predictors of the primary outcome, while drain removal before 15 days predicted lymphocele significantly (B=-2.4, p<0.000).

Conclusions

LDRT has specific risk factors for lymphatic complications, which may be related to extent of recipient vascular dissection, arterial anastomotic time, and early drain removal.

## Introduction

The high incidence of non-communicable diseases like diabetes mellitus and hypertension in India has led to an epidemic of chronic kidney disease (CKD). More than 200,000 CKD patients need renal transplants every year. Despite India being the second largest transplanting country in the world in terms of the absolute number of RTs (~8000 of 95,000 global), there is a huge shortfall leading to the addition of more than 100,000 CKD patients to the transplant waiting list every year [1]. The deceased donor renal transplant (DDRT) to living donor renal transplant (LDRT) ratio is only 0.24 and compares unfavorably with the developed countries [2]. Infrastructure and funding for RT are also suboptimal in most public hospitals, resulting in long waiting lists and long hemodialysis to renal transplant intervals, even for LDRTs. 

Lymphatic complications after renal transplant are well-documented in literature. The incidence of lymphocele following renal transplant varies from 5-33% [3]. The suspected risk factors studied are age, BMI, fluid overload, hemodialysis period, diabetes, presence of acute rejection, arterial anastomosis type, DDRT, use of steroids or diuretics, and coagulopathy [4-8]. Most of these studies are retrospective and deal with DDRTs. This is partly because they are based on western data where the LDRT rate is much lower (vide supra) The lymphatic complication noted in most of these studies is a lymphocele, which is a clinically or radiologically documented lymphatic collection.

As mentioned earlier, most of our renal transplants are LDRTs. Unexpectedly, we have found the rate of lymphatic complications (LCs) in our unit to be high (>15%). These LCs include not only lymphoceles but also prolonged lymphatic output from the postoperative drains. The LCs are a significant source of recipient morbidity and prolonged hospital stay. We undertook this prospective study in our renal transplant patients to study the risk factors of lymphocele as well as prolonged lymphatic drainage. There is an existing lack of such prospective data in contemporary literature, especially from India.

## Materials and methods

This prospective analytical study was conducted in the renal transplant unit of our tertiary care hospital in north India over a period of three years. The study was cleared by the institutional ethics committee (No. TP (48/2018)/IEC/PGIMER/RMLH/1881/18 dated October 24, 2018), as per the Declaration of Helsinki guidelines. Written informed consent was obtained from all the participants. The evaluation of prospective LDRT pairs was performed as per standard protocols; hematological and biochemical screening, blood grouping and human leukocyte antigen (HLA) typing, and screening for viral illnesses (HIV, hepatitis B surface antigen, hepatitis C virus, cytomegalovirus) and common malignancies (breast, prostate, lung, cervix, etc.) were performed. Co-morbid conditions like hypertension, diabetes, coronary artery disease, and hyperthyroidism were optimized as per guidelines. In the second stage of evaluation, i.e. after the donor’s status was deemed safe and legally valid, donors underwent CT angiography of the renal vessels, nuclear isotope scan, diethylene triamine penta-acetic acid (DTPA) scan for individual kidney function, and crossmatch for B and T lymphocytes including DNA typing. The recipient underwent duplex imaging to assess the iliofemoral vessels for atheroma or thrombus. The relationship between the donor and recipient was documented. The recipients who did not have an eligible or fit for living donor were listed for DDRT as per national guidelines issued by the National Organ and Tissue Transplant Organization (NOTTO) [2]. All demographic and clinical details of the enrolled transplant pairs were recorded in the case record forms.

The renal transplant procedure was performed by a standard technique; both open and laparoscopic donor nephrectomy patients were enrolled. The graft anastomoses were extraperitoneal (preferably right) with an end-to-side graft arterial to external iliac artery anastomosis, and a modified Lich-Gregoir type of ureteroneocystostomy. Other anastomotic techniques were selected in specific circumstances. An extraperitoneal drain was placed near the graft and removed on the fifth postoperative day or later, with an output of less than 50 mL/day. Urinary leak was ruled out by serial triglyceride and creatinine levels of the drain fluid. The operating team was unchanged in all the cases. Meticulous hilar dissection was done with electrocautery, both in the donor and in the perivascular area of the recipient, to minimize lymphatic leak. All the variables expected to affect the primary outcome measure like age, gender, etiology of CKD, relationship, induction regimen, duration of pre-operative dialysis, warm ischemia time (anastomotic time), and HLA mismatch ≥3 were recorded. In the postoperative period, routine duplex scans were done on the third postoperative day. Other imaging studies like USG or CT scans were guided by clinical indications like falling output, limb/perineal edema, fever, unexplained pain or hypertension, prolonged drainage, and others. A standard triple immunosuppression protocol was followed both for induction and maintenance, using parenteral methylprednisolone (oral prednisolone later), oral tacrolimus (0.1mg/kg/day), and oral mycophenolate mofetil (1.5- 2g/day). Parenteral basiliximab, anti-thymocyte globulin, or plasmapheresis was used in high immunological risk scenarios like spousal donors, HLA mismatch >4, recent transfusion, or deceased donor transplantation. The total dose of parenteral methylprednisolone did not exceed 1.5g. The minimum follow-up period was six months.

For the purpose of the study, delayed graft function (DGF) was defined as the need for postoperative hemodialysis and/or serum creatinine>2 in the postoperative period analyzed at week 1; while graft dysfunction at 90 days was defined as supra-normal serum creatinine values (>1.2 mg/dL). Prolonged lymphatic drainage (PLD) was defined as an inability to remove the drain till postoperative day 15 due to output ≥50 mL/day. Lymphocele was defined as a clinically significant (visible swelling, falling urine output, limb/perineal edema, fever, unexplained pain or hypertension, and prolonged drainage) or radiologically-detected collection at least 100mL around the graft after postoperative day 15 and deemed responsible for the experienced symptoms/signs. The primary outcome measure was taken as the presence of either PLD or lymphocele. 

Statistical Analysis

Data were analyzed using IBM®SPSS Statistics® Trial version 2019-20 (IBM, Chicago, USA). Descriptive statistics were used for clinical and demographic characteristics; mean and standard deviation were used if the data were normally distributed, otherwise median was preferred. For univariate analytical statistics, chi-square test and t-test were used for qualitative and quantitative variables, respectively. Confidence intervals were taken within 95% and a significant p-value was <0.05. To identify risk factors for the primary outcome measure and lymphocele, binary logistic regression (simultaneous and forward stepwise method) was used with the outcome parameter as the dependent variable. The B co-efficient determined the extent of percentage change caused by the risk factor on the dependent variable. The efficacy of the model was verified by standard criteria like Omnibus tests of model coefficients, log-likelihood ratio, and Nagelkerke’s R-square method.

## Results

During the three-year period of the study, a total of 165 LDRT pairs reported for transplant. Of these, 11 pairs were excluded due to the recipient being declared unfit for surgery, two donors had unfavorable vascular anatomy, and two recipients died whilst awaiting surgery. Hence, 150 pairs were included. The male:female ratio of recipients was ~4:1, while it was 1:4 for donors. The clinical and demographic parameters have been detailed in Table 1. Laparoscopic donor nephrectomy was performed in 11 of 145 donors (7.6%). Most recipients (143/150) underwent end-to-side graft artery to external iliac artery anastomosis. 

**Table 1:** Demographic and clinical factors in 150 patients undergoing renal transplant and evaluated for lymphatic complications (univariate analysis) All continuous variables depicted in terms of mean ± standard deviation; categorical variables depicted by number (percentage); difference in means analyzed using t-test, while difference in proportions analyzed using chi-square test. *A p-value of 0.05 was statistically significant. ESRD: End-stage renal disease; MPO: Methylprednisolone parenteral for induction; ATG: Anti-thymocyte globulin for induction; POD: postoperative day; HLA: Human leukocyte antigen

Factor	Primary outcome negative (no lymphocele/ drain output for <15 days) n=109 (72.67%)	Primary outcome positive (lymphocele +/ drain output for ≥15 days) n=41 (27.3%)	p value
Mean age of recipients (years)	32.37 ± 9.98	34.29 ± 12.38	0.33
Mean age of donors (years)	42.96 ± 10.32	44.64 ± 11.42	0.40
Male gender (recipient)	87 (79.8%)	31 (20.7%)	0.57
Male gender (donor)	21 (19.8%)	7 (4.8%)	0.80
ETIOLOGY of ESRD			
Diabetic nephropathy	32 (29.4%)	14 (34.1%)	0.31
Hypertensive nephropathy	28 (25.7%)	6 (14.6%)	
IgA nephropathy	15 (13.8%)	2 (4.9%)	
Chronic glomerulonephritis	10 (9.2%)	5 (12.2%)	
Autosomal dominant polycystic kidney disease	3 (2.8%)	3 (7.3%)	
Alport’s syndrome	4 (3.7%)	2 (4.9%)	
Focal segmental glomerulosclerosis	10 (9.2%)	3 (7.3%)	
Minimal change disease	7 (6.4%)	6 (14.6%)	
RELATIONSHIP OF DONOR WITH RECIPIENT			
Mother	41 (37.6%)	15 (36.6%)	0.85
Father	11 (10.1%)	6 (14.6%)	
Wife	34 (31.2%)	13 (31.7%)	
Sibling	14 (12.8%)	4 (9.8%)	
Son	1 (0.9%)	1 (2.4%)	
Husband	5 (4.6%)	0	
Unrelated (deceased donor)	3 (2.8%)	2 (4.9%)	
GENETIC RELATIONSHIP			
Related	67 (61.5%)	26 (63.4%)	0.85
Unrelated	42 (38.5%)	15 (36.6%)	
ABO incompatible transplant	5 (4.6%)	3 (7.3%)	0.68
HLA mismatch ≥3	31 (28.4%)	11 (26.8%)	0.85
Mean HLA mismatch between pairs	3.15 ± 1.40	3.05 ± 1.39	0.65
Deceased donor renal transplant (DDRT)	3 (2.8%)	2 (4.9%)	0.6
Living donor renal transplant (LDRT)	106 (97.2%)	39 (95.1%)	0.6
Mean duration of pre-transplant hemodialysis (months)	19.7 ± 21.5	14.8 ± 15.3	0.18
IMMUNOSUPPRESSANT INDUCTION REGIMEN			
MPO	67 (61.5%)	25 (61%)	0.96
MPO + Basiliximab	34 (31.2%)	13 (31.7%)	0.95
MPO ± ATG	5 (4.6%)	1 (2.4%)	0.89
MPO ± plasmapheresis	3 (2.8%)	2 (4.9%)	0.89
Mean Warm Ischemia Time (WIT in minutes)	53.8 ± 13.5	50.9 ± 11.6	0.24
Acute rejection	11 (10.1%)	9 (22%)	0.06
Delayed graft function (DGF) POD7	27 (24.8%)	23 (56.1%)	<0.001*
Graft dysfunction POD 90	11 (10.1%)	10 (24.4%)	0.02*

Lymphocele and prolonged drainage >15 days

A total of 32 of 150 patients (21%) developed lymphocele. The lymphocele was diagnosed by persistent serous wound discharge in 10 of 32 patients (31%), limb or perineal edema in seven (21%), oliguria in five (16%), hypertension in five (16%), and, incidentally detected on radiology in five (16%). The mean time to diagnosis postoperatively was 32 days. Another nine patients required prolonged usage of the postoperative drain, making the proportion of primary outcome patients 41 of 150 (27%). Ultrasonography was used to confirm the diagnosis of lymphocele in all patients with clinical suspicion and a CT scan was performed in 17 of the 41 patients (41%); contrast was administered if the serum creatinine values were below 1.3 mg/dL. Two patients had simultaneous graft artery stenosis, which was managed by percutaneous angioplasty and stenting. Of the 41 patients with lymphatic complications, 25 (60%) were managed with either continued drainage or stoma bag placement over the drainage/ leakage site. Eight patients (20%) underwent formal USG-guided drainage of the collection, while seven (17%) had laparoscopic fenestration procedures. Operative drainage was considered if the collection diameter was more than 5cm or there was obstruction of the vessels or ureter. All the laparoscopic procedures were successful and there was no recurrence over six months of follow-up. The mortality in the study group was three out of 150 (2%); none of the deaths were attributable to lymphatic complications. One of the deaths was caused by bleeding secondary to early percutaneous intervention for graft artery stenosis. None of the patients were administered sclerosant therapy for the lymphatic complications. Three patients who developed lymphoceles and two who had prolonged drainage had kidneys transplanted after laparoscopic donor nephrectomy; however, due to the low proportion of the cases of laparoscopy and a large number of variables, the variables of laparoscopic donor nephrectomy and end-to-side external iliac artery (ES EIA) anastomosis were not included in the regression model.

Regression analysis

The DDRT: LDRT ratio was 5:145 or 0.03; most donors were genetically related to the recipient (93 of 150, 62%). As seen in Table 1, only DGF on the seventh postoperative day (p<0.001) and graft dysfunction at 90 days (p=0.02) were significantly associated with the primary outcome (lymphocele and/or prolonged drain output >15 days) on univariate analysis using chi-square and t-tests. Nine of 150 (6.2%) donor nephrectomies were performed laparoscopically. Table 2 depicts simultaneous logistic regression analyses of risk factors for both lymphocele and the primary outcome measure, where all the independent variables were entered simultaneously in the model regression equation. Warm ischemia time (WIT, p=0.034), DGF at POD7 (p=0.004) and drain removal after 15 days (p<0.000), were associated with lymphocele. When the dependent variable was lymphocele and/ or drain removal after 15 days (the primary outcome), the same factors had significant association with the dependent variable (WIT, p=0.039, and DGF at POD7, p=0.011). None of the other factors were significantly associated with the dependent variable.

**Table 2:** Factors predictive of lymphocele formation and primary outcome (lymphocele ± drain output ≥ 15 days) using logistic regression analysis (simultaneous method) in 150 patients *A p-value of 0.05 was statistically significant. ESRD: End-stage renal disease; MPO: Methylprednisolone parenteral for induction; ATG: Anti-thymocyte globulin for induction; POD: postoperative day; HLA: Human leukocyte antigen

	Lymphocele				Lymphocele ± drain output ≥ 15 days			
Factor	B coefficient	Standard error (B)	Wald coefficient	p-value	B coefficient	Standard error (B)	Wald coefficient	p-value
Mean age of recipients (years)	0.021	0.05	0.17	0.68	0.076	0.044	2.99	0.084
Mean age of donors (years)	0.011	0.05	0.05	0.83	-0.029	0.039	0.54	0.46
Recipient gender	-1.45	0.786	3.41	0.065	-0.45	0.59	0.59	0.44
Donor gender	1.58	1.41	1.26	0.26	-0.40	1.15	0.12	0.73
ETIOLOGY of ESRD			5.83	0.56			7.60	0.37
Diabetic nephropathy	-1.12	0.95	1.39	0.24	-0.53	0.78	0.45	0.50
Hypertensive nephropathy	-1.36	1.01	1.83	0.18	-0.99	0.85	1.35	0.26
IgA nephropathy	-1.42	1.38	1.06	0.30	-1.998	1.14	3.07	0.080
Chronic glomerulonephritis	-0.31	1.10	0.08	0.78	0.296	0.94	0.099	0.75
Autosomal dominant polycystic kidney disease	1.22	1.51	0.66	0.42	1.06	1.26	0.71	0.40
Alport’s syndrome	-1.42	1.72	0.68	0.41	-0.49	1.21	0.17	0.69
Focal segmental glomerulosclerosis	-0.086	1.19	0.005	0.94	-0.42	1.02	0.17	0.68
RELATIONSHIP OF DONOR WITH RECIPIENT			3.85	0.57			2.05	0.84
Mother	-1.41	3.63	0.15	0.697	0.79	2.94	0.071	0.79
Father	-4.44	3.75	1.40	0.24	1.65	2.91	0.32	0.57
Wife	3.14	2.85	1.21	0.27	-0.25	2.06	0.015	0.903
Sibling	-1.48	3.17	0.219	0.64	-0.58	2.55	0.052	0.82
Husband	-20.95	14130.38	0.000	0.999	-19.84	16775.86	0.000	0.999
Genetic relationship present	1.80	3.89	0.22	0.64	0.67	2.93	0.053	0.82
Mean HLA mismatch between pairs	-0.40	0.46	0.76	0.39	0.24	0.35	0.46	0.496
HLA mismatch ≥3	0.35	1.05	0.12	0.73	0.65	0.797	0.66	0.42
ABO incompatible transplant	-0.45	1.46	0.095	0.76	-0.73	0.98	0.56	0.46
Mean duration of pre-transplant hemodialysis (months)	-0.033	0.022	2.26	0.13	-0.024	0.016	2.18	0.14
IMMUNOSUPPRESSANT INDUCTION REGIMEN			5.11	0.16			0.72	0.87
MPO	-1.53	2.04	0.56	0.45	-1.35	1.83	0.54	0.46
MPO + Basiliximab	-3.24	1.91	2.89	0.089	-1.29	1.73	0.56	0.45
MPO ± ATG	-3.22	2.43	1.76	0.18	-1.27	1.72	0.54	0.46
Mean warm ischemia time (WIT in minutes)	-0.066	0.031	4.49	0.034*	-0.045	0.022	4.28	0.039*
Acute rejection	-0.75	0.87	0.74	0.39	-0.44	0.68	0.42	0.52
Delayed graft function (DGF) POD7	-2.37	0.83	8.19	0.004*	-1.54	0.60	6.52	0.011*
Graft dysfunction POD 90	1.73	0.97	3.197	0.074	-0.101	0.73	0.02	0.89
Drain removal after POD 15	-3.40	0.79	18.52	<0.000*	-	-	-	-

Table 3 depicts logistic regression using the same independent and dependent variables as in Table 2, however, here the forward stepwise regression model is used to exclude independent predicting variables in steps. Hence, lymphocele was significantly associated with DGF at POD7 (p=0.010), WIT (p=0.044), and drain removal after 15 days (p<0.000). The primary outcome variable was significantly associated with DGF at the seventh postoperative day (p<0.000) and WIT (p=0.024). The robustness of both the regression models was confirmed by Omnibus tests of model coefficients, log-likelihood ratio, and Nagelkerke’s R-square method. Factors that were not significantly associated in any of the regression models were age and gender of the donor and recipient, etiology of CKD as a whole or individually, individual relationship or genetic relationship, ABO incompatibility, type of donor nephrectomy, LDRT or DDRT, duration of pre-operative hemodialysis, HLA mismatch, type of induction immunosuppression, and acute rejection.

**Table 3:** Factors predictive of lymphocele formation and primary outcome (lymphocele ± drain output ≥ 15 days) using logistic regression analysis (multinomial, forward stepwise method) in 150 patients *A p-value of 0.05 was statistically significant. ESRD: End-stage renal disease; MPO: Methylprednisolone parenteral for induction; ATG: Anti-thymocyte globulin for induction; POD: postoperative day; HLA: Human leukocyte antigen

	Lymphocele				Lymphocele ± drain output ≥ 15 days			
Factor	B coefficient	Standard error (B)	Wald coefficient	p-value	B coefficient	Standard error (B)	Wald coefficient	p-value
Delayed graft function (DGF) POD 7	-1.27	0.497	6.55	0.010*	-1.69	0.42	15.87	<0.000*
Drain removal after POD 15	-2.4	0.55	18.80	<0.000*	-	-	-	-
Mean duration of pre-transplant hemodialysis (months)	-0.015	0.014	1.230	0.267	-	-	-	-
Mean warm ischemia time (WIT in minutes)	-0.04	0.02	4.05	0.044*	-0.038	0.017	5.06	0.024*

## Discussion

Lymphocele is a frequent and troublesome complication after renal transplant, occurring in 0.6-33.9% of cases [4,9-12]. It can be variously attributed to surgical and medical predisposing factors. Amongst the surgical factors, perivascular dissection leading to disruption of lymphatics is probably the most contributory. Though a few studies have suggested dissection during donor nephrectomy as the primary cause of LCs [7,13], most authors agree that recipient dissection of the iliac bed is the main cause of LCs [3,4,6,14]. It is therefore suggested to ensure meticulous ‘lymphostasis’, for which no particular technique amongst electrocoagulation, ultrasonic shears, or suture ligation has proven superior [15,16]. We perform lymphatic closure with bipolar electrocautery and suture ligation, wherever necessary. Since donor nephrectomy is less contributory for LCs, the technique of donor nephrectomy (open or laparoscopic) also does not differ in this regard, as shown by Tefik et al. in 58 patients [13]. Supernumerary arteries or the type of arterial anastomosis (end-to-end internal iliac artery (EE IIA) versus ES EIA) are also not significant risk factors for LCs [17,18], though Inoue et al. identified ES EIA as a risk factor [6]. We performed only 11 laparoscopic donor nephrectomies during the study period. Despite this, our LC rate was more than 20%, emphasizing the probable contribution of medical factors or recipient operative steps. Since almost all our anastomoses were ES EIA with a wide dissection of the iliac bed, we have to reassess our technique in this regard. The significant association of warm ischemia time (anastomotic time) in our study also warrants a relook at existing techniques. 

In a recent review, Ranghino and colleagues [10] summarized the etiology and management of post-transplant lymphoceles. The established medical factors were diabetes mellitus [9], high doses of steroids or mycophenolate or diuretics or crystalloids [5,19,20], prolonged pre-operative hemodialysis [21], delayed graft function or acute rejection, coagulopathy, obesity, [12,14] and warm ischemia time [19]. Many of these factors predispose to lymphorrhea due to continued inflammation inside or around the graft with exudation [13]. The most consistent risk factors in our study by all statistical models were the warm ischemia time and delayed graft function. As mentioned earlier, this may be associated with suboptimal techniques of dissection and arterial anastomosis earlier in our experience. In our mostly genetically-related LDRT population, other medical factors did not show significant association, probably due to standard protocols followed. Of the 150 patients, 20 had acute rejection; however, the association with LC was not significant (p=0.06). Anti-thymocyte globulin (ATG) and basiliximab were used only in selected cases, which did not show any association. We did not use high-dose steroids (except for rejection), mammalian target of rapamycin inhibitors (mTOR)-sirolimus, or everolimus in any patient. The BMI of our patients was also low, as they were from modest socioeconomic backgrounds.

Lymphocele or lymphorrhea as the primary outcome?

Most studies assessing LCs in renal transplant have used lymphocele as a primary outcome measure for elucidation of risk factors. In our experience, lymphorrhea or prolonged drainage is part of the same spectrum and needs more documentation, due to its effect on hospital stay and morbidity. This has been advocated by other authors. Saidi et al. demonstrated prolonged drainage for recipients receiving laparoscopic nephrectomy grafts [7]. Inoue et al. and Tefik et al. advocate assessment of lymphatic complications by observation of the prolonged lymphatic drainage. They also opine that extended drain placement alone can prevent many lymphoceles, rendering other sclerosant methods like povidone-iodine superfluous [6,13]. The only challenge is prolonged hospital stay. Our results indicate a significant association between early drain removal and subsequent lymphocele. Since ours is a public hospital, prolonged hospital stay is not an individual financial concern. Patients can also be safely discharged with a stoma bag after removal of the drain. Sclerosant therapy has not been proven to be effective or safe universally, and we do not practice it [6,22].

The major highlight of our study and what it adds to existing literature is the prospective data it provides and the fact that it analyses risk factors in living donors. The sample size also compares to other similar studies (Table 4). As seen in Table 4, most authors have analyzed retrospective data and deal with deceased donors. Different risk factors are relevant in different settings. We believe that factors in a deceased donor graft may differ from living donor transplants. The cadaveric kidney is hurriedly mobilized and dissected, leaving the possibility of open lymphatics. High doses of steroids and ATG are also administered to these patients, as are large volumes of resuscitation fluids. All these factors are known to predispose to lymphatic complications. Some authors have emphasized the importance of continued drainage, even if inconvenient [6]. We have successfully used a stoma bag collection in the event of prolonged drainage; it mitigates the risk of infection. The drain opening has not stenosed significantly in our experience after removal of the drain. Hence, while looking for predictors in the LDRT setting, we should study predominantly LDRT patients. The established treatment of lymphocele more than 5cm or symptomatic is laparoscopic fenestration, with a recurrence rate of less than 10%. The treatment of lymphorrhea or wound leakage is less successful. The focus of future research is mainly preventive techniques, the most promising of which appears to be prophylactic peritoneal fenestration [23,24]. We have also planned a study on the same in the near future.

Limitations of our study

Though our study has 150 patients, a larger sample size would be more conclusive for logistic regression analysis. More number of laparoscopic cases would also make our data more contemporary, as laparoscopic donor nephrectomy is a preferred modality whenever feasible. Since our patients are from low-resource settings with unfavourable pre-transplant factors, our DGF rates are higher. This can be improved by enhanced capacity-building, mainly in the public sector. 

**Table 4:** Recent studies evaluating the causes of post-renal transplant lymphocele LDRT: Living donor renal transplant; DDRT: Deceased donor renal transplant

S. No.	Authors [reference], year	Number of patients/ prospective or retrospective	LDRT/DDRT	Number of lymphatic complications	Risk factors	Treatment offered and suggested preventive strategies
1.	Saidi et al. [7], 2009	642/ retrospective over eight years	297 DDRT; 127 open nephrectomy (OP); 218 laparoscopic nephrectomy (LP)	DDRT: 5.4 ± 0.7 days of drainage; LP: 8.6 ± 2.7 days; OP: 5.6 ± 1.2 days	Use of ultrasonic shears in LP group	Back dissection and meticulous ligation, or open ligation of lymphatics
2.	Bzoma et al. [8], 2016	740/ retrospective over seven years	All DDRT	Lymphoceles requiring treatment in 59 (8%)	Recipient age, acute rejection, delayed graft function	Open (53) and laparoscopic (6) fenestration; prompt identification of risk factors essential
3.	Tefik et al. [13], 2015	58/ retrospective comparison between open and laparoscopic donor nephrectomy over two years	All LDRT	Three lymphoceles in the laparoscopic group. Duration of drainage in cranial (~6 days) and caudal (~9 days) not different between groups	Longer drainage time during acute rejection episodes	Percutaneous drainage of one lymphocele; suggested bench ligation of lymphatics and adipose tissue
4.	Inoue et al. [6], 2017	244/ retrospective over 15 years	All LDRT (open, laparoscopic, hand-assisted donor nephrectomies mixed)	40 (16.4%) persistent lymphatic leaks, 10 (4.1%) lymphoceles	End-to-side graft to external iliac artery anastomosis	Puncture of lymphocele in 10 patients, continued drainage till amount less than 50 ml, no sclerosants or fenestration
5.	Ulrich et al. [9], 2009	426/ retrospective over four years	DDRT 366, LDRT 60	42 lymphoceles, 24 needed surgery	Diabetes (mainly), tacrolimus therapy, acute rejection	Laparoscopic fenestration in 26 (if size>5 cm/ symptomatic), percutaneous intervention in eight patients
6.	Dubeaux et al. [4], 2004	450/ retrospective over 13 years	All DDRT	3 (0.6%) lymphoceles	Not analyzed	Open fenestration in one, percutaneous intervention in two. Suggested meticulous lymphatic ligation in donor and recipient, and adequate postoperative open drainage
7.	Heer et al. [5], 2017	250/ retrospective data recorded prospectively over eight years	DDRT 138 (55%), LDRT 112 (45%)	31 lymphoceles (12.4%)	No significant risk factor identified, studied fluid resuscitation as a factor	Open fenestration in three, laparoscopic fenestration in 11, percutaneous intervention in five. Suggested low steroid immunosuppression, optimum fluid therapy
8.	Our study	150/ prospective over three years	LDRT 145 (97%); DDRT 5 (3%)	41 (27%) patients with lymphatic complications; 32 lymphoceles, 9 prolonged drainage > 15 days	Warm ischemia time, delayed graft function at seven days, removal of drain before 15 days	25 (60%) continued drain or stoma bag, eight (20%) underwent ultrasound-guided percutaneous drainage, seven (17%) had laparoscopic fenestration. We suggest optimum recipient bed dissection for iliac vessels, anastomotic time to be kept as low as possible, drain or stoma bag to be kept till minimal output

## Conclusions

There are specific risk factors in LDRT for lymphatic complications, which may be related to extent of recipient vascular dissection, arterial anastomotic time, and early drain removal. Usage of a stoma bag in cases of prolonged drainage is a satisfactory alternative. A larger sample size, as well as performance of more laparoscopic donor nephrectomies, which are almost a standard-of-care, would allow for better evaluation of the risk factors. It is prudent to consider prolonged drainage postoperatively as a lymphatic complication, after ruling out urinary leaks. Extended drain placement avoids future surgery for lymphocele without any significant added complications.
